# Long Non-Coding RNA Expression Profile Associated with Malignant Progression of Oral Submucous Fibrosis

**DOI:** 10.1155/2019/6835176

**Published:** 2019-07-29

**Authors:** Shanghui Zhou, Yun Zhu, Zhijing He, Dahe Zhang, Feng Guo, Xinchun Jian, Chenping Zhang

**Affiliations:** ^1^Department of Oral & Maxillofacial - Head & Neck Oncology, Shanghai Ninth People's Hospital, College of Stomatology, Shanghai Jiao Tong University School of Medicine, Shanghai 200011, China; ^2^National Clinical Research Center for Oral Diseases, China; ^3^Shanghai Key Laboratory of Stomatology & Shanghai Research Institute of Stomatology, China; ^4^Department of Oral and Maxillofacial Surgery, the Second Xiangya Hospital, Central South University, Changsha, Hunan 410011, China; ^5^Department of Oral and Maxillofacial Surgery, Xiangya Hospital, Central South University, Changsha, Hunan 410008, China

## Abstract

Oral submucous fibrosis (OSF) as one of the premalignant disorders endures a series of histopathological stages to invasive oral squamous cell carcinoma (OSCC) eventually. However, the role of long non-coding RNA (lncRNA) expression in OSF malignant progression still remains poorly understood. Through RNA-sequencing normal mucous, OSF and OSCC tissues, we found 687 lncRNA transcripts significantly and differentially expressed during OSF progression, including 231 upregulated lncRNAs and 456 downregulated lncRNAs, indicating that lncRNAs are involved in the regulation of different stages of OSF development. Further functional enrichment analysis showed these differentially expressed lncRNAs participated in inflammation signaling, Wnt signaling, angiogenesis, CCKR signaling, integrin signaling, PDGF signaling, p53 signaling, and EGF receptor (EGFR) signaling pathways, which contribute to inflammatory and fibroelastic pathogenetic changes of OSF and further malignant progression. Five novel lncRNAs were differentially expressed during OSF progression with varied expression levels, indicating the importance of these lncRNAs in OSF malignant development. Moreover, some lncRNAs have been previously identified to be associated with OSCC pathogenesis, including* HCG22, RP11-397A16.1, LINC00271, CTD-3179P9.1,* and* ZNF667-AS1*. Thus, our study firstly comprehensively elucidated lncRNAs expression profile of malignant procession from OSF premalignant lesion to OSCC, which will enlighten our understanding of the importance of lncRNA involved in OSF malignant development.

## 1. Introduction

Oral squamous cell carcinoma (OSCC) is one of the most common types of malignant tumors in head and neck [1]. OSCC has the characteristics of rapid progress, wide infiltration range, easy cervical lymphatic metastasis and poor prognosis [2, 3]. OSCC patients have a lower rate of early visits (< 50%), which directly impede enhancement of 5-year survival rate. Oral submucous fibrosis (OSF), as one of the pre-cancerous lesions in OSCC [4], is a chronic, occult oral mucosal disease associated with chewing betel nut [5]. OSF can be divided into early, middle and late stages pathologically, which may be three steps during OSF developing OSCC [6]. Notably, malignant transformation rate of OSF has been increasing with range from 3% to 19% [6, 7]. Therefore, identification of molecular markers for early detection and diagnosis of OSF malignant progression is urgent.

Long non-coding RNAs (lncRNAs), as RNA transcripts longer than 200 nucleotides lacking an open reading frame (ORF), play an important role in multiple biological processes including gene transcription, post-transcriptional regulation, splicing/modification and regulation of protein synthesis [8]. Recent RNA-sequencing based transcriptome studies demonstrated that 68% of human transcripts are lncRNAs, and approximately 80% of them are unannotated [9]. More importantly, the deregulation and dysfunction of lncRNAs have been implicated in a variety of malignancies including cancer [10]. lncRNAs are differentially expressed in multiple human tumor tissues compared with normal tissues, and involved in cell transcription, chromatin remodeling and vascular invasion as an oncogenes or tumor suppressor genes [11].

Several studies elucidating the role of lncRNAs in OSCC tumorigenesis have been performed. Through analyzed SAGE libraries of normal oral mucosa and premalignant lesions, 195 differentially expressed lncRNAs have been identified to be involved in the early stages of OSCC development [12]. Analysis of existing microarray data from OSCC and normal oral mucosa samples showed a series of differentially expressed lnRNAs involved in ECM-receptor interaction, inflammation-related functions and Toll-like receptor signaling pathways [13]. Through analysis of TCGA dataset, 728 lncRNA differentially expressed transcripts were found by RNA-sequencing in human head and neck squamous cell carcinoma (HNSCC), and some of them were associated with HPV infection [14]. These studies strongly suggest that lncRNAs play an important role in OSCC initiation and progression, which will provide novel insight into the potential of lncRNAs as biomarkers and therapeutic targets in OSCC. However, little is known about the significance and biological function of lncRNAs during OSF malignant progression.

Here, we characterized differentially expressed lncRNAs in three stages during normal-OSF-OSCC development using RNA-sequencing. We established the link between lncRNAs and their target genes using the ‘cis' and ‘trans' mode, elucidated significantly changed biological processes and signaling pathways during OSF progression. We also verified a set of differentially expressed lncRNAs in OSF development. Overall, we obtained the first comprehensive expression profile of lncRNA across developmental stages of OSCC, which provides a new insight into the molecular and cellular events associated with lncRNAs during OSF malignant progression.

## 2. Materials and Methods

### 2.1. Sample Collection

Fresh tissue specimens of OSCC, OSF, and normal oral mucosa were obtained at the time of surgical resection at the Second Xiangya Hospital and Xiangya Hospital, Central South University (Changsha, China) and Shanghai Ninth People's Hospital, Shanghai Jiaotong University School of Medicine (Shanghai, China) from January 2016 to June 2017. The patients' informed consents had been obtained under a protocol reviewed and approved by the Institutional Review Boards of the Xiangya School of Medicine or Shanghai Jiaotong University School of Medicine. Two normal oral mucosa specimens (ON1, ON2) were obtained from 2 healthy individuals. Eight OSCC samples (T) were from 8 patients diagnosed with squamous cell carcinoma of the oral cavity, and their adjacent tissues were collected as OSF samples (N, OM). OSF is diagnosed excluding OSCC or neoplastic disease. All of examined specimens were pathologically confirmed by two pathologists independently. Archived RNA from independent cohorts include 13 normal mucous samples, 10 OSF samples, and 20 OSCC samples [15, 16].

### 2.2. Total RNA Isolation

Total RNA was isolated using TRIzol reagent (MRI, USA), and then measured by using Nanodrop 2.0.

### 2.3. Library Preparation for lncRNA Sequencing

Epicentre Ribo-ZeroTM kit was used to remove rRNA. Subsequently, the fragmentation buffer was used to break the RNA into short fragments of 150-200 bp, which was used as a template to synthesize cDNA using random hexamers. After purification, the double-stranded cDNA was subjected to terminal repair; a tail was added and the sequencing linker was ligated, and finally, cDNA library of total RNA was obtained by PCR enrichment. After the library was constructed, preliminary quantification was performed using Qubit2.0, and the library was diluted to 1 ng/ul. Then, the insert size of the library was detected using Agilent 2100. After the insert size was as expected, the effective concentration of the library was accurately quantified using the qPCR method (Library effective concentration > 2 nM) to ensure library quality. After the library was qualified, the different libraries were pooled according to the effective concentration and the target data volume, and then sequenced by Illumina HiSeq (E-GENE Co., Ltd, Shenzhen, China).

### 2.4. Data Analysis

The original sequencing sequence contains low-quality Reads with connectors. To ensure the quality of information analysis, Raw Reads must be filtered to obtain Clean Reads to ensure the reliability of subsequent analysis data. Raw Reads were filtered and aligned to the reference genome by alignment software HISAT and then compared the ratio of the genome on the alignment and the distribution of Reads on the chromosome. Reference genome version is hg19, and the annotation file is from the Genecode database, version v19.

### 2.5. Novel lnRNAs Prediction

The results of the sample alignment using the Hisat software are first assembled by stringtie (version stringtie-1.3.3b). Subsequently, the assembly results of the respective samples were further combined and compared with the known gene set, the minimum transcript set was obtained by preliminary filtering (filtering conditions: number of exons >=2, transcript length >= 200 bp, covering reads >=3, the expression amount FPKM (fragments per kilobase of exon model per million reads mapped) >=0.5). Based on the assembly results, according to the structural characteristics of lncRNA and the functional characteristics of non-encoded proteins, a series of strict screening conditions were set, and the selected lncRNA was chose as the final candidate lncRNA set for subsequent analysis. The coding ability of transcripts was predicted to distinguish between mRNA and lncRNA. Three software programs (CPC, IncRScan, and CNCI, respectively) were used to make predictions score coding ability of the transcript and then set a scoring threshold. Finally, new lncRNAs were confirmed when the three predictive software consistently judged to be non-coding transcripts (E-GENE Co., Ltd, Shenzhen, China).

### 2.6. Quantitative Analysis

Through the analysis of the above two parts, the known transcripts and novel lncRNAs were integrated, all subsequent transcript sets were obtained for analysis and quantified, and the FPKM method was used to efficiently and accurately calculate the expression amount of each gene by the Python-based toolkit HTseq.

### 2.7. GO and PANTHER Pathway Enrichment Analysis

The quantification of both lncRNAs and coding genes in each sample was calculated by Cuffdiff (v2.1.1)27, and transcripts with a* P*-adjust < 0.05 were assigned as being differentially expressed. PANTHER pathway is a database resource for understanding high-level functions and utilities of the biological system; we thus examined the statistical enrichment of differential expression genes or lncRNA target genes in PANTHER pathways (http://www.pantherdb.org/pathway/).

### 2.8. Real-Time RT-PCR

RNA samples were further analyzed by qPCR. Total cDNA was synthesized using GoScript™ Reverse Transcription System (Promega, WI, USA). qPCR was performed using Promega™ GoTaq™ qPCR Master Mix (Promega, WI, USA) according to manuscript's protocol.

All amplifications were followed by dissociation curve analysis of the amplified products. Specific primers were designed, specificities were confirmed by BLAST in NCBI, and gene expression levels were normalized with* GAPDH*. The correlation between the results of RNA-Seq and qPCR was calculated using correlation test.

### 2.9. Statistical Analysis

Statistical analysis was carried out using the R software package (http://cran.r-project.org/). Differences between the expression levels of lncRNAs in normal mucous, OSF, and OSCC tissues were evaluated by paired t-test.* P* <0.05 was considered significant. The Pearson correlation coefficient and R function cor() were used to compute a correlation matrix.

## 3. Results

### 3.1. Genome-Wide Discovery of lncRNAs in OSF Malignant Progression

**Figure 1:**
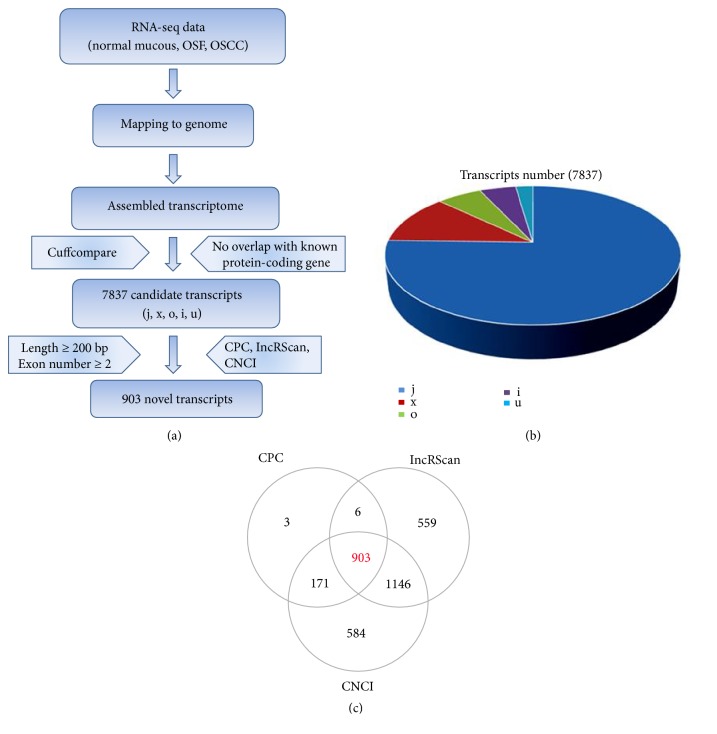
Characterization of novel lncRNAs during OSF progression to OSCC. (a) The schematic diagram for identifying pipeline for lncRNAs of normal mucous, OSF and OSCC, with a detailed description in the Materials and Methods. (b) Screening of candidate lincRNA, intronic lncRNA, anti-sense lncRNA type transcripts by comparison with known mRNAs and using class_code information in cuffcompare analysis results (screening class_code for i, j, u, x or o transcription). (c) Identification of novel lncRNAs by using CPC, IncRScan, and CNCI. 903 novel transcripts were selected using three software evaluated protein-coding transcripts and remove putative protein-coding transcripts. j: potentially novel isoform, as least one splice junction is shared with a reference transcript; i: intronic transcript; u: unknown intergenic transcript; o: generic exonic overlap with a reference transcript; x: natural antisense transcript, NAT; i: intronic transcript.

To comprehensively identify the lncRNA landscape in OSF malignant progression, we performed lncRNA expression profile of 2 normal mucous tissues, 8 OSF tissues with different stages and 8 OSCC combined with OSF tissues using RNA sequencing. A total of ~1.5-2.0 billion clean reads of each sample were obtained after mapped to the human reference genome using hierarchical indexing for spliced alignment of transcripts (HISAT) (Table S1; Figure 1(a)), of which more than 74% of the average reads were mapped to the human reference genome and more than 40% of the average reads were uniquely mapped to the genome (Table S2).

To distinguish lncRNA candidates, a highly stringent pipeline with 5 hierarchical filtering steps was employed (Figure 1(a)). The filter removed short, unreliable transcripts homology with non-lncRNA and non-mRNA (e.g., rRNA, tRNA, snRNA, snoRNA, pre-miRNA, pseudogenes, etc.) and was further compared with known mRNAs and used the class-code information in the cuffcompare analysis results (screen class_code for transcription of i, j, u, x, or o transcripts). Furthermore, the CPC, lncRScan, and CNCI were used to assess protein-coding potential to remove potential coding transcripts (Figures 1(b) and 1(c)). Thus, a total set of 7,837 transcripts (including 903 novel lncRNAs) were obtained and defined candidate lncRNA during OSF malignant progression (Table S2).

**Figure 2:**
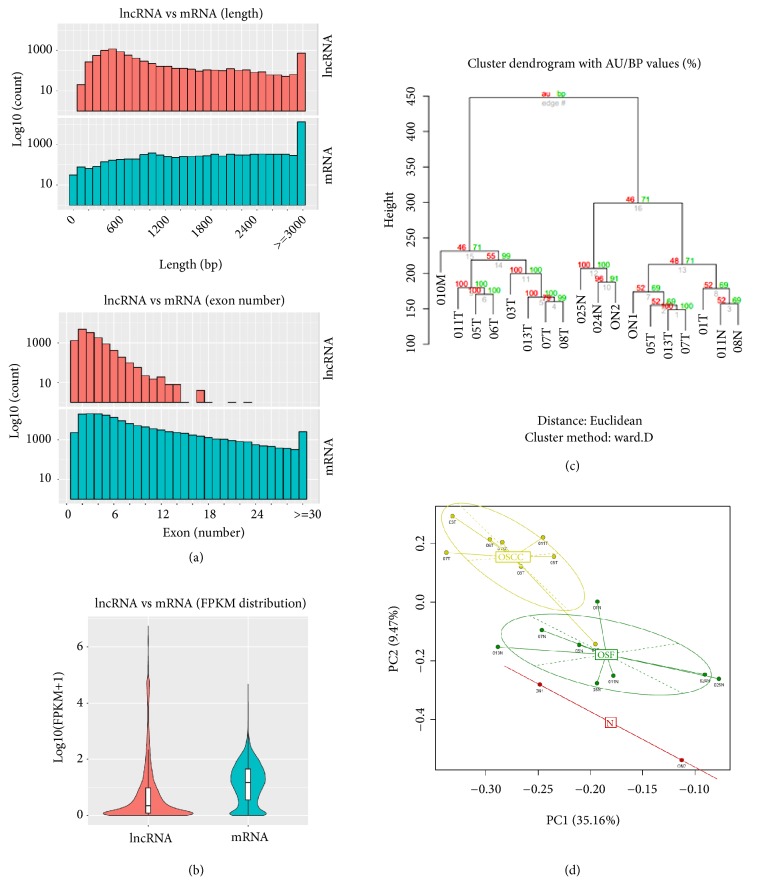
Structure comparison of lncRNAs and mRNAs in the malignant progression of OSF. Distribution of the number of (a) length and exons, as well as (b) FPKM of lncRNAs and mRNAs. (c) The cluster analysis of expression levels of transcripts in 18 tissue samples (2 normal mucous, 8 OSF and 8 OSCC samples). (d) PCA diagram shows separate expression pattern during OSF progression to OSCC, through analyzing lncRNA expression levels in normal mucous, OSF and OSCC samples, localized to Normal (red), OSF (green) and OSCC (yellow).

We further quantitatively analyzed expression levels of lncRNAs for read counts and FPKM. Results showed that the lengths of most of lncRNAs were between 200 bp and 800 bp, while the lengths of most of mRNAs were over 3,000 bp (Figure 2(a)). Moreover, most of lncRNAs contained 2 exons, while most of mRNAs contained over 2 exons (Figure 2(a)). We also evaluated expression levels between lncRNAs and mRNAs groups (Figure 2(b)). Results showed that the average lncRNA expression level was lower than the mRNA average expression level.

### 3.2. Identification of Differentially Expressed lncRNAs in OSF Malignant Progression

The expression levels of lncRNA and protein-coding transcripts in normal mucous, OSF tissues, and OSCC combined with OSF samples were quantified by FPKM using Cuffdiff. A cluster dendrogram of the expressed genes showed clearly separation among normal mucous, OSF tissues, and OSCC tissues by significant changes in the pattern of differentially expressed transcripts (Figure 2(c)). Principal component analysis (PCA) consistently showed the variance among normal mucous, OSF and OSCC tissues (Figure 2(d)).

**Figure 3:**
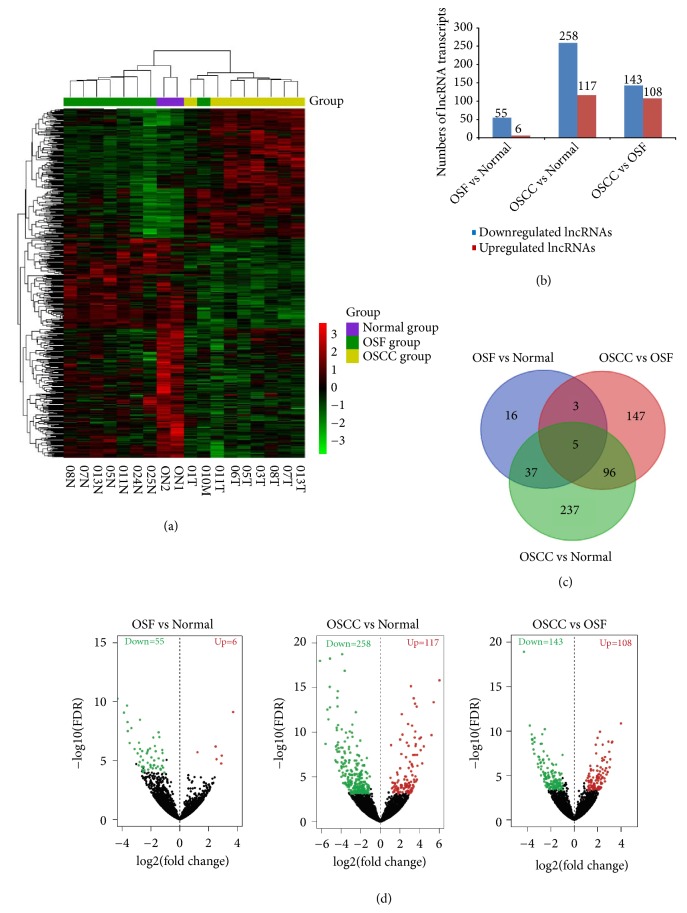
Genome-wide profiling of differentially expressed lncRNAs during OSF progression to OSCC. (a) The hierarchical heat map showing different expression pattern of lnRNAs. Red represents relatively high expression and the green represents relatively low expression. (b) Numbers of differently expressed lncRNA transcripts as shown by the histogram. (c) Venn diagram and (d) volcano plot showing differentially expressed lncRNAs in three comparison groups (OSF vs normal, OSCC vs normal and OSCC vs OSF).

A hierarchical clustering was constructed and clearly showed differentiated lncRNA expression pattern during OSF malignant progression (Figure 3(a)). We identified 687 lncRNA transcripts that were significantly differentially expressed during OSF malignant progression (fold change > 2,* P *< 0.05) (Table S3), including 231 upregulated lncRNAs and 456 downregulated lncRNAs (Figure 3(b)). To further analyze the interactions among the differentially expressed lncRNAs, we constructed a Venn diagram using differentially expressed transcripts in normal mucous, OSF tissues and OSCC combined with OSF samples (Figure 3(c), Table S4). We identified 5 differentially expressed lncRNAs (*RP11-172F10.1, RP1-34H18.1, RP11-276H19.1, RP11- 466L17.1, RP11-707A18.1*) detected among all three compared groups ( OSF-vs-Normal; OSCC-vs-Normal; OSCC-vs-OSF). 8 differentially expressed lncRNAs (*RP11-1082L8.3, RP11-108M12.3, RP11-172F10.1, RP11-276H19.1, RP11-314N14.1, RP11-466L17.1, RP11-707A18.1 *and* RP1-34H18.1*) were found in groups of OSF-vs-Normal and OSCC-vs-OSF. However, stage-specific differentially expressed lncRNAs were not detected. We also found that the number of downregulated lncRNAs was higher than the number of upregulated lncRNAs during OSF malignant progression, which were also demonstrated in volcano plots (Figure 3(d)). These results indicate the involvement of differentially expressed lncRNAs in OSF malignant progression.

### 3.3. Functional Enrichment of Differentially Expressed lncRNA Target Genes during OSF Progression

**Figure 4:**
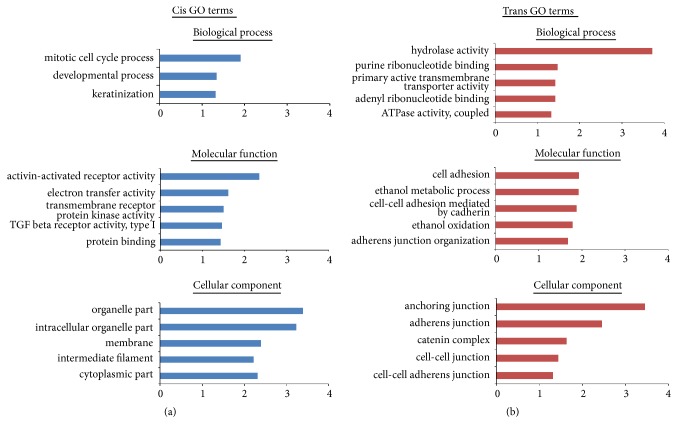
Functional prediction of target genes regulated by differentially expressed lncRNAs. GO enrichment histogram showing (a) cis and (b) trans interactions targeted by lncRNA.

**Figure 5:**
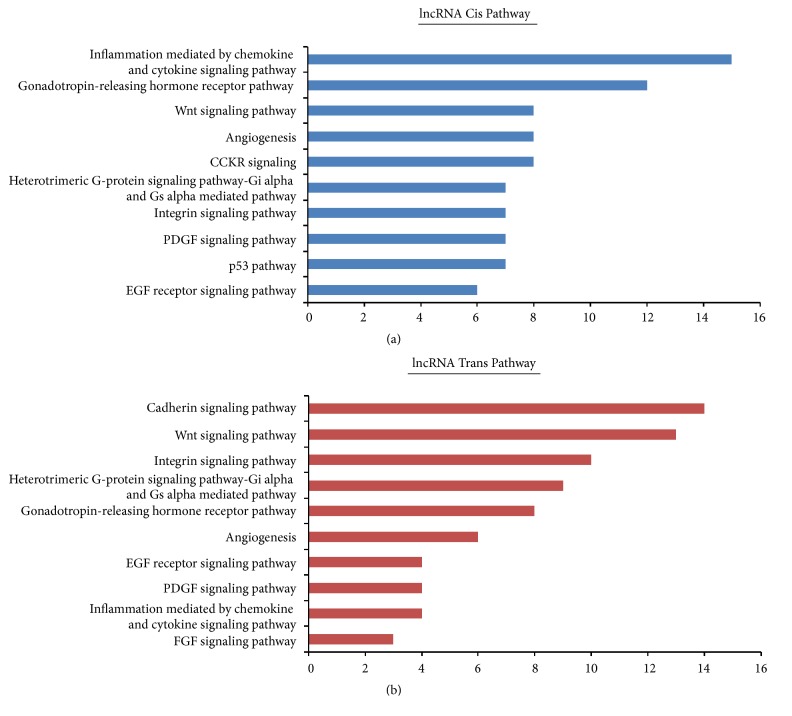
PANTHER pathway analysis of (a) cis- and (b) trans-target genes of differential expressed lncRNAs. Enriched pathways with significant p-values are shown.

lncRNA function is mainly achieved by acting on protein-encoding target genes through cis or trans. To investigate the possible functions of potential target genes of lncRNAs in cis- and trans-regulatory relationships during OSF progression, we analyzed relevant GO terms and pathways. The protein-coding genes near upstream and downstream 100 kb of the lncRNA loci were selected as cis-target genes [17]. We found 1094 lncRNAs transcribed close to 8494 cis-target genes, while 588 cis-target genes are near to 540 differentially expressed lncRNAs during OSF progression (Table S5). GO analysis showed differentially expressed GO terms in the OSF progression, including mitotic cell cycle, developmental and keratinization processes involved in biological process (Figure 4(a)). PANTHER pathway analysis showed top 10 pathways closely related OSF progression, including inflammation mediated by chemokine and cytokine signaling pathway, gonadotropin-releasing hormone receptor pathway, Wnt signaling pathway, angiogenesis, CCKR signaling pathway, heterotrimeric G-protein signaling pathway-Gi alpha and Gs alpha-mediated pathway, integrin signaling pathway, PDGF signaling pathway, p53 pathway and EGF receptor (EGFR) signaling pathway (Figure 5(a)).

The 1292 trans-target genes of 1093 lncRNAs are defined by correlations between lncRNA and mRNA expression levels (Table S6) [18], and 557 trans-targets from differentially expressed lncRNA during OSF malignant progression were identified. The top enriched terms included hydrolase activity and ribonucleotide binding in biological processes; cell adhesion and ethanol metabolic process in molecular function; anchoring junction and adherens junction in cellular component (Figure 4(b)). Pathway analysis further showed top 10 enriched pathways including cadherin signaling pathway, Wnt signaling pathway, integrin signaling pathway, heterotrimeric G-protein signaling pathway-Gi alpha and Gs alpha-mediated pathway, gonadotropin-releasing hormone receptor pathway, angiogenesis, EGFR signaling pathway, PDGF signaling pathway, Inflammation mediated by chemokine and cytokine signaling pathway, and FGF signaling pathway (Figure 5(b)).

### 3.4. Validation of Differentially Expressed lncRNAs during OSF Progression

**Figure 6:**
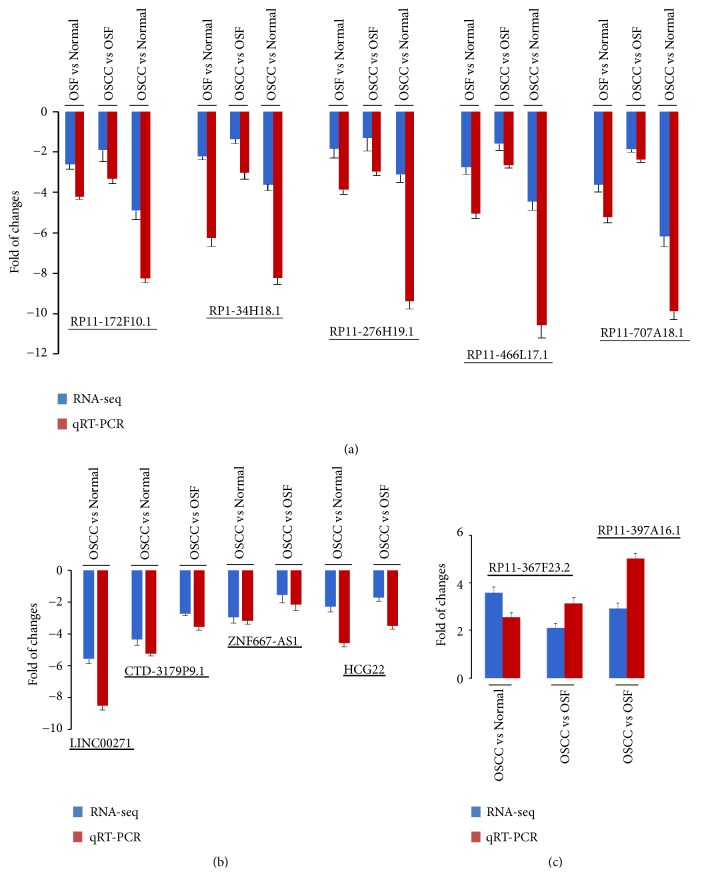
Comparison results between qRT-PCR and RNA sequencing. Eleven lncRNAs were selected to further validate in an independent cohort of 13 normal mucous samples, 10 OSF samples, and 20 OSCC samples by qRT-PCR assay. The expression levels of 11 downregulated and upregulated lncRNAs were concordant with RNA-Seq results.

We selected five differentially expressed lncRNAs in all the stages of OSF progression and examined their expression patterns by qPCR. Results showed that these five lncRNAs (*RP11-172F10.1, RP1-34H18.1, RP11-276H19.1, RP11-466L17.1, *and* RP11-707A18.1*) were significantly downregulated at OSF and OSCC tissues compared to normal mucosa tissues (Figure 6(a), Table S7), which are consistent with the expression patterns from the RNA-Seq data. We further selected several known and novel lncRNAs to verify, including 4 downregulated lncRNAs (*LINC00271, CTD-3179P9.1 ZNF667-AS1, *and* HCG22*), and 2 upregulated lncRNAs (*RP11-367F23.2, *and* RP11-397A16.1*) in groups of OSCC-vs-Normal and/or OSCC-vs-OSF (Figure 6(a), Table S7). These results suggest that lncRNAs expressions are consistent with RNA-sequencing data in the malignant progression of OSF.

## 4. Discussion

Emerging evidence has showed lncRNAs play a critical role in oral tumorigenesis. In this study, we assessed genome-wide lncRNA expression profile by next-generation sequencing to unravel the potential role of lncRNAs during OSF progression. We found that 687 differentially expressed lncRNAs were shown among normal mucous, OSF and OSCC samples, including 231 up-regulated and 456 down-regulated lncRNAs. Further functional investigation revealed cis-target genes and trans-target genes related to differentially expressed lncRNAs during these three stages, and their biological functions. Pathways analysis showed deregulated signaling pathways during OSF progression, including inflammation, Wnt signaling, angiogenesis, integrin signaling, PDGF signaling, p53 signaling, and EGFR signaling pathways. We also verified several newly identified lncRNAs during the malignant progression of OSF, which might contribute to OSF development through deregulating their target genes.

Recent studies demonstrated that lncRNAs are correlated with lymph node metastasis of OSCC [19–21], indicating that lncRNA deregulation plays a critical role in OSCC development. OSF as a precancerous stage is caused by juxta-epithelial inflammatory reaction followed by progressive fibroelastic changes of the submucosal tissues. Our data confirmed some uncharacterized lncRNAs involved in inflammation signaling pathway mediated by chemokine and cytokine during OSF progression, such as RP5-1011O1.3, CXorf49, RAMP2-AS1, RP11-932O9.9, and MSTRG.11710.1. We also confirmed several signaling pathways significantly related to fibroblast biology and fibrosis deregulated by lnRNAs in OSF progression. For example, RP11-875H7.5, RP11-714L20.1, and RP11-879F14.2 deregulate Wnt/*β*-catenin signaling pathway; RP1-261G23.5 and LINC00551 are involved in angiogenesis signaling pathway; MSTRG.17280.2 and MSTRG.17417.1 participated in integrin signaling pathway. Further investigation of biological functions and underlying mechanisms of these novel lncRNAs is needed.

In our study, we also found some lncRNAs that have been identified to be involved in OSCC pathogenesis by other groups, further supporting the accuracy of our study, such as* HCG22, RP11-397A16.1, LINC00271, CTD-3179P9.1 *and* ZNF667-AS1 (MORT). *For example, downregulation of* HCG22 *and* CTD-3179P9.1* was related to increased overall survival of HNSCC patients, while RP11-397A16.1 upregulation was associated with decreased overall survival of HNSCC patients [14, 22].

ZNF667-AS1 was highly expressed in human normal cells but reduced in immortalized cells and multiple tumor tissues due to promoter CpG methylation, suggesting its importance in the early stage of carcinogenesis [23, 24]. ZNF667-AS1 suppressed cell proliferation, migration, and invasion ability of laryngeal squamous cell carcinoma cells [25]. Abnormally expressed lncRNA was detected in OSCC metastatic tissue samples and saliva samples, suggesting that detection of lncRNA can be used as a tool for non-invasive, early and rapid diagnosis of OSCC [26].

Thus, this study demonstrated the comprehensive lncRNA expression map during OSF progression. A series of newly identified lncRNAs with or without known functions have been identified. Understanding the precise molecular mechanisms caused by lncRNAs will be a critical step in exploring epigenetic etiology of OSF and OSCC pathogenesis, which provides us with potential epigenetic biomarkers for OSF and OSCC early detection in future. Our study on the potential link between lncRNAs and three OSF different development stages presents a novel area for further investigations into the target genes of these novel lncRNAs, leading to develop therapeutic strategies for OSF and OSCC eventually.

## Data Availability

RNA-Seq data generated in this study are deposited in the Gene Expression Omnibus (GEO) database under the accession numbers ‘‘GSE125866”.

## Supplementary Materials

Supplementary Materials
* Supplementary Table S1.* Summary of RNA-seq data and reads mapped to the human reference genome.* Supplementary Table S2.* List of lncRNAs identified during the OSF progression to OSCC.* Supplementary Table S3.* List of differentially expressed lncRNAs during the OSF progression to OSCC.* Supplementary Table S4.* List of differentially expressed lncRNAs among three stages of OSF progression.* Supplementary Table S5.* List of cis-acting lncRNAs during the OSF progression to OSCC.* Supplementary Table S6.* List of tans-acting lncRNAs during the OSF progression to OSCC.* Supplementary Table S7.* Primers used in the qPCR analysis.
